# Thermogenic Adipose Redox Mechanisms: Potential Targets for Metabolic Disease Therapies

**DOI:** 10.3390/antiox12010196

**Published:** 2023-01-14

**Authors:** Ashley K. Putman, G. Andres Contreras, Emilio P. Mottillo

**Affiliations:** 1 Department of Large Animal Clinical Sciences, College of Veterinary Medicine, Michigan State University, 784 Wilson Road, East Lansing, MI 48823, USA; 2 Hypertension and Vascular Research Division, Henry Ford Hospital, 6135 Woodward Avenue, Detroit, MI 48202, USA; 3 Department of Physiology, Wayne State University, 540 E Canfield St., Detroit, MI 48202, USA

**Keywords:** adipose tissue, thermogenesis, metabolic disease, redox reactions

## Abstract

Metabolic diseases, such as diabetes and non-alcoholic fatty liver disease (NAFLD), have several negative health outcomes on affected humans. Dysregulated energy metabolism is a key component underlying the pathophysiology of these conditions. Adipose tissue is a fundamental regulator of energy homeostasis that utilizes several redox reactions to carry out the metabolism. Brown and beige adipose tissues, in particular, perform highly oxidative reactions during non-shivering thermogenesis to dissipate energy as heat. The appropriate regulation of energy metabolism then requires coordinated antioxidant mechanisms to counterbalance the oxidation reactions. Indeed, non-shivering thermogenesis activation can cause striking changes in concentrations of both oxidants and antioxidants in order to adapt to various oxidative environments. Current therapeutic options for metabolic diseases either translate poorly from rodent models to humans (in part due to the challenges of creating a physiologically relevant rodent model) or tend to have numerous side effects, necessitating novel therapies. As increased brown adipose tissue activity results in enhanced energy expenditure and is associated with beneficial effects on metabolic health, such as decreased obesity, it has gathered great interest as a modulator of metabolic disease. One potential reason for the beneficial health effects may be that although non-shivering thermogenesis is enormously oxidative, it is also associated with decreased oxidant formation after its activation. However, targeting its redox mechanisms specifically to alter metabolic disease remains an underexplored area. Therefore, this review will discuss the role of adipose tissue in energy homeostasis, non-shivering thermogenesis in adults, and redox mechanisms that may serve as novel therapeutic targets of metabolic disease.

## 1. Introduction

Metabolic diseases, such as diabetes and non-alcoholic fatty liver disease (NAFLD), have serious implications for human health. For instance, afflicted patients suffer from an increased risk of developing cancer, nephropathy, and cardiovascular diseases [1,2]. The estimated incidence of NAFLD alone is 25% in the United States, imposing economic costs of approximately USD 103 billion on the health care system [3]. Although of great concern to the medical community, the pathophysiology of metabolic diseases is complicated by numerous underlying mechanisms, presenting a challenge in terms of fully understanding and treating these diseases.

One well-established underlying component of metabolic disease pathophysiology is the dysregulated energy metabolism [4,5]. A key attribute of adipose tissue to maintain energy homeostasis is its specialized properties to both store and expend energy. Whether accumulation or dissipation pathways are activated, many highly coordinated biochemical oxidation and reduction (redox) reactions must take place for the proper metabolic regulation by adipocytes [6,7]. As a result of these metabolic redox reactions, reactive metabolites are formed and are crucial for normal cellular function but are also capable of causing substantial damage. Therefore, mammals have developed numerous antioxidant mechanisms to maintain the redox balance and prevent damage by unchecked reactive metabolites [8]. Among the fat cells in adipose depots, brown adipocytes in particular have an especially high capacity for oxidative reactions during non-shivering thermogenesis (NST), relying on an appropriate oxidant–antioxidant balance to carry out their function. For instance, brown adipose tissue (BAT) may effectively support NST by limiting antioxidant pathways that could block thermogenic mechanisms while still maintaining reactive metabolites within a non-toxic range [9,10,11]. However, these dynamics vary in differing metabolic states, such as lean versus obese [9].

The vital role of BAT in regulating energy expenditure has made this tissue a desirable target for metabolic disease therapies [12,13]. Indeed, BAT is associated with improved cardiometabolic health [14]. However, many treatments currently available are riddled with several side effects. For instance, drugs such as mirabegron promote NST but can have off-target effects on the cardiovascular system, leading to heart failure [15,16]. Thus, there is a need to develop novel therapeutics that effectively mitigate these diseases without adverse outcomes. The integral role of redox mechanisms to NST and the vast evidence of disrupted redox regulation in metabolic disorders suggest there may be an opportunity to exploit these pathways to accomplish this aim. We will discuss the role of adipose tissue in maintaining whole-body energy homeostasis, NST and the redox systems in place to carry out this crucial function, and how the intrinsic mechanisms of thermogenic fat to avoid excessive oxidation may be manipulated as a therapy for metabolic disease. Although non-adipocytes cells can also be a significant source of ROS, this review will particularity focus on the adipocyte and mechanisms within [17,18].

## 2. Adipose Tissue as a Modulator of Energy Homeostasis

### 2.1. Adipose Depots and Lipolysis

Within the last several decades, it has become clear that adipose tissue functions beyond a passive energy storage organ. Indeed, different adipose depots support whole-body energy homeostasis in a highly regulated, dynamic manner and influence other metabolically active organs via the secretion of molecules such as hormones and fatty acids [19,20,21]. There are two primary depots of adipose tissue, white (WAT) and BAT, with a form of recruitable brown adipocyte also possible (i.e., beige/brite adipose). White adipocytes are characterized by a peripherally located nucleus and a single lipid droplet. During times of energy excess, WAT accumulates fatty acids and stores them as triglycerides. In energy shortages, triglycerides are released via lipolysis to be used as fuel by other organs [22,23]. Brown adipocytes, in contrast, are evolved to be a cell of energy expenditure. These cells have a centrally located nucleus with multiple, small lipid droplets. Additionally, BAT is characterized by numerous mitochondria [24]. The abundance of mitochondria and the mitochondrial membrane protein uncoupling protein 1 (UCP1; discussed below) make BAT highly specialized in dissipating energy as heat during NST [25].

Although both BAT and beige adipose perform NST, the development of these thermogenic fats can differ [26]. One pathway for thermogenic fat formation is de novo adipogenesis, which is the process of preadipocyte differentiation to either brown or beige fat [27,28]. Alternatively, the direct conversion of WAT to beige adipose can occur, whereby mature white adipocytes transdifferentiate into beige adipocytes after NST stimulation [26]. Interestingly, these beige adipocytes are present in newly weaned mice and in term infants which can readily be recruited with vast UCP1 expression in adult mice during cold exposure or β-adrenergic stimulation [29,30,31]. Thus, beige adipocytes are highly plastic and share characteristics of both WAT and BAT [29].

Each adipose depot must intricately balance triglyceride storage and release. If lipid metabolism becomes dysregulated, fatty acids may accumulate in non-adipose tissues and cause lipotoxicity [32]. Indeed, the overaccumulation of lipids is associated with several metabolic diseases including diabetes and fatty liver disease (FLD) [33]. Thus, promoting energy expenditure is a highly desirable pathway to combat excessive lipid accumulation [12,13]. One such pathway is lipolysis, in which adipocytes facilitate the release of energy in the form of fatty acids by providing substrates for beta-oxidation. It is well-established that β-adrenergic stimulation is the primary inducer of lipolysis in rodent adipose tissue [34,35]. Traditionally, this pathway is activated by catecholamines, particularly norepinephrine. However, lipolysis can also be triggered by other molecules, including, but not limited to, cytokines, lipid mediators (e.g., eicosanoids), glucocorticoids, endotoxin, and thyroid hormones [23,36,37,38]. Stimulating β-adrenergic receptors leads to a series of interactions and intracellular changes, reviewed in detail elsewhere, that lead to the release of fatty acids by lipases [35,39]. Specifically, patatin-like phospholipase domain containing-2 (also known as adipose triglyceride lipase), hormone sensitive lipase, and monoacylglycerol lipase work in tandem to release free fatty acids from triglycerides [40,41,42]. Fatty acids released from lipolysis can be utilized in a myriad of ways, including cellular signaling and oxidation for ATP generation [39]. Importantly, as discussed below, the mechanisms of energy dissipation provide a sink for these released substrates to be oxidized.

### 2.2. Beta-Oxidation and Coupled Respiration

Fatty acids destined for mitochondrial beta-oxidation must first be transported into the cell with facilitation by specific proteins such as CD36. Once inside the cell, fatty acids are activated to acyl-coenzyme A (acyl-CoA) esters by acyl-CoA synthetases [43]. Subsequently, acyl-CoA must be imported into the mitochondria for fatty acid oxidation, which requires the carnitine cycle [43]. However, in certain tissues such as the heart, exogenous fatty acids must first cycle through lipid droplets [44,45]. Beta-oxidation occurs inside the mitochondrion, utilizing four enzymatic steps in a cyclical process: oxidation, hydration, second oxidation, and thiolysis. [43,46]. An acetyl-CoA molecule, the fuel for the tricarboxylic acid cycle and ketogenesis, is formed with each cycle. Additionally, reduced nicotinamide adenine dinucleotide (NADH) and flavin adenine dinucleotide (FADH_2_) are formed to be used in the respiratory chain (alternatively, the electron transport chain; ETC) [43].

**Figure 1:**
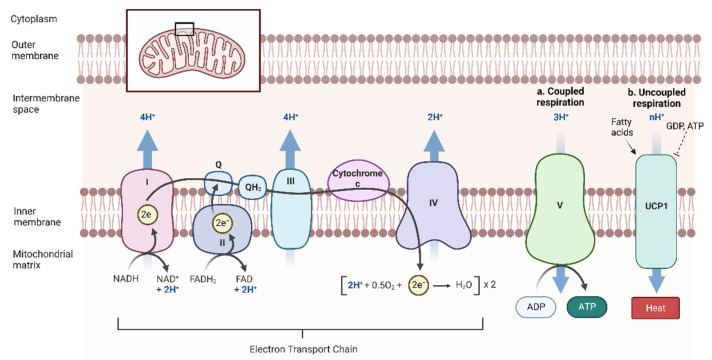
Coupled and uncoupled respiration in thermogenic adipose tissues. Both coupled and uncoupled respiration occur in the mitochondria (inset). Following free fatty release and beta−oxidation, reducing equivalents nicotinamide adenine dinucleotide (NADH) and flavin adenine dinucleotide (FADH_2_) are produced to be used in the electron transport chain. Electrons subsequently move through four enzymes (Complexes I–IV) and two mobile electron carriers (ubiquinone, Q, and cytochrome c) within the inner mitochondrial membrane, which results in protons moving to the intermembrane space. Movement of the aforementioned protons through Complex V back into the mitochondrial matrix is coupled to ATP production (**a**). During thermogenesis, uncoupling protein 1 (UCP1) is induced and promotes backflow of protons across the inner membrane. Protonic backpressure is reduced, leading to maximally accelerated NADH and FADH_2_ formation, and oxidation is consequently uncoupled from phosphorylation, resulting in heat production instead of ATP generation (**b**). H^+^ = proton; e^−^ = electron; Q = ubiquinone; QH_2_ = reduced ubiquinone, ubiquinol. Figure created with biorender.com.

The ETC is composed of five enzymes embedded within the inner mitochondrial membrane, and two mobile electron carriers that function to transfer energy through a series of redox reactions [47]. NADH and FADH_2_ are oxidized, donating electrons to Complex I (NADH-ubiquinone oxidoreductase) and II (succinate-ubiquinone oxidoreductase) of the ETC, respectively [48]. The donated electrons subsequently transfer to ubiquinone (Q), reducing it to ubiquinol (QH_2_) [48]. Complex III (ubiquinol-cytochrome c oxidoreductase) then oxidizes QH_2_, moving electrons to cytochrome c [48]. Transport to Complex IV (cytochrome c oxidase) occurs next, where oxygen serves as the final electron acceptor and is reduced to water [48]. This also results in a net of two protons moving into the intermembrane space, contributing to a proton motive force. The movement of these protons back to the mitochondrial matrix via Complex V is coupled to ATP production (Figure 1a) [48]. In BAT and beige adipose specifically, fatty acids can be used as fuel through the ETC or as allosteric regulators of UCP1 [39]. Indeed, BAT mitochondria have a high capacity for fatty acid oxidation, producing reducing equivalents that simultaneously drive ATP generation and energy dissipation after UCP1 activation [49]. Given its superior ability to expend energy, NST has garnered substantial research attention as a treatment for metabolic diseases [12,13].

### 2.3. Thermogenesis

Adaptive thermogenesis is the process an organism adopts to generate heat upon various environmental cues. Classically, there are two types of thermogenesis: shivering and NST [49]. The most relevant environmental stimuli that induce thermogenesis include cold exposure and exorbitant caloric intake, although the latter has been debated [50,51]. These cues promote β-adrenergic signaling and lipolysis, which result in the release of fatty acids as discussed above. Although the focus of this review is β-adrenergic-dependent NST, it should be noted that β-adrenergic-independent mechanisms (e.g., nicotinic acetylcholine receptor signaling) have been reported in beige adipocytes from inguinal WAT [52].

Once stimulated, NST can proceed through multiple mechanisms. Since the 1970s, it has been demonstrated that UCP1 is the dominant protein responsible for thermogenesis induced during cold exposure [53]. Therefore, the main mechanisms of NST are broadly classified as either UCP1-dependent or UCP1-independent. In contrast to ATP production during coupled respiration, UCP1 allows for the futile cycling of protons and backflow across the inner mitochondrial membrane. Consequently, oxidation becomes separated from the oxidative phosphorylation necessary for energy production in a phenomenon termed “uncoupled respiration.” As a result, the proton gradient across the inner mitochondrial membrane of thermogenic fat is decreased, which can be inhibited by purine nucleotides such as GDP and ATP [54]. The resulting loss of protonic backpressure leads to NADH and FADH_2_ generation at an enhanced rate [49]. Although respiration is accelerated in this case, the energy of the respiratory chain is released as heat rather than being directed towards ATP production (Figure 1b) [55]. Thus, uncoupled respiration during NST is capable of expending substantial amounts of energy.

Although once thought to be indispensable, some pivotal studies provided evidence that UCP1 is not needed for NST in all instances [56,57,58,59]. Mechanisms that are independent of UCP1 include calcium cycling, glycerol-3-phosphate shuttle activation, lipid turnover, and creatine metabolism [49]. Indeed, congenic mice lacking UCP1 were less tolerant of acute cold exposure than wild type mice, but were able to adapt to and resist cold when the temperature gradually declined [56]. The authors additionally found that these mice needed to consume more oxygen (i.e., perform less efficient thermogenesis) compared to wild type mice to acclimate [56]. One proposed mechanism supported in this study was increased calcium cycling in beige adipocytes [56]. The cycling of fat oxidation and fatty acid synthesis during chronic adrenergic stimulation could contribute to the energy expenditure in adipose tissue independently of UCP1. Fatty acid synthesis can refer to both the formation of new triglycerides (de novo lipogenesis) or the re-esterification of fatty acids that were previously hydrolyzed. Either situation can facilitate energy expenditure because both processes consume ATP and when coupled to oxidation, provide a potential energy sink [60]. Indeed, Mottillo and colleagues found that the genes of both fatty acid oxidation and triglyceride synthesis were upregulated across all depots of adipose tissue during chronic β3-adrenergic stimulation; however, these effects were largely independent of UCP1 expression [60]. Creatine is also a major regulator of thermogenesis. During creatine cycling, mitochondrial ATP is used to generate phosphocreatine from creatine, releasing large molar amounts of ADP, which can then stimulate cellular respiration. Phosphocreatine then interacts with a phosphatase, regenerating creatine that is available to continue the cycle [57]. In murine beige adipocytes, creatine metabolism was upregulated and enhanced the energy expenditure via ATP turnover when UCP1-dependent thermogenesis was lacking [57]. Decreasing creatine synthesis reduced diet-induced thermogenesis in mice, an effect most prominent in brown adipocytes, although beige adipocytes were affected as well [61]. Finally, skeletal muscle also utilizes calcium and creatine cycling, and thus represents another tissue that performs NST [62,63]. Regardless of the pathway, the regulation of systemic energy metabolism in thermogenic fat tissue requires highly coordinated redox reactions carried out by interdependent reactive metabolites and antioxidant molecules [9].

## 3. Metabolic Redox Reactions in Thermogenic Adipose Tissue

### 3.1. Role of Reactive Metabolites

Thermogenic adipose tissues direct fatty acids through the redox reactions necessary for metabolic programs such as ATP generation and NST. Although coupled respiration in adipose ETC is highly efficient, not all electrons are used for ATP generation. Indeed, premature leaking of some electrons at Complexes I, II (to a lesser extent), and III is a common phenomenon resulting in the formation of highly reactive molecules known as reactive oxygen species (ROS) [48]. Furthermore, adipocytes produce reactive nitrogen species (RNS), which can also act on the mitochondria and interact with ROS to form additional reactive metabolites [64]. Common examples of these chemically reactive species in biological systems include lipid peroxides, superoxide anion (O_2_^●−^), hydroxyl radical (OH^●^), hydrogen peroxide (H_2_O_2_), nitric oxide (NO^●^), and peroxynitrite (ONOO−).

Adipose tissue is comprised of a heterogenous population of cells, including adipocytes, preadipocytes, fibroblasts, and immune cells [65]. Of the non-adipocytes, macrophages can also contribute to the redox balance [17]. For instance, adipose resident macrophages are a major source of ROS [66]. Within adipocytes, sources of reactive metabolites include the mitochondria, cytosol, endoplasmic reticulum, peroxisomes, phagosomes, and the plasma membrane [18]. Mitochondrial energy production is well-established as the primary site of intracellular ROS production [48,67]. Leaked electrons from the ETC result in the formation of O_2_^●−^, which can subsequently form molecular oxygen and the less reactive H_2_O_2_ through dismutation [67]. Importantly, O_2_^●−^ is the parent of most ROS, including the highly reactive OH^●^ and ONOO− [68]. Of note, the sheer abundance of mitochondria in thermogenic adipocytes means that it is not only thermogenic mechanisms, but also the increased capacity for coupled respiration that can contribute to the ROS and RNS pool [69,70]. A non-exhaustive list of associations between reactive metabolites and thermogenic fat tissues is shown in Table 1.

While NST is highly oxidative and could reasonably be associated with an increase in ROS production, enhanced uncoupling activity is generally paired with a decrease in their formation [78,79]. Therefore, it is unsurprising that conflicting evidence exists in regard to the relationship between NST and ROS production. Shabalina and coauthors found that ROS production in BAT mitochondria was affected by UCP1 activity under exogenous succinate supplementation, but not in conditions that were more physiologically relevant (e.g., endogenous succinate, glycerol-3-phosphate, acyl-CoA, or pyruvate substrate addition) [80]. They concluded that it is unlikely for ROS production to be curtailed by a rise in UCP1 activity [80]. In contrast, Chouchani and colleagues recently provided a detailed in vivo description of ROS dynamics during acute NST in BAT that suggests thermogenesis increases reactive metabolite production [71]. Upon cold exposure, mouse mitochondrial O_2_^●−^, lipid hydroperoxides, and H_2_O_2_ were increased in BAT in vivo. Furthermore, the authors elaborated the mechanism in which ROS contributed to thermogenesis, determining that UCP1 cysteine 253 was sulfenylated, thus altering sensitivity to activation of the UCP1-dependent NST [71]. Later work provided by the same laboratory identified succinate as a driver of UCP1-dependent NST in BAT due to the increased ROS production via oxidation of succinate dehydrogenase [81]. The discordant results between studies may be due to differences in experimental design and conditions, such as the substrate used for mitochondrial respiration [80].

Although often thought of as intrinsically deleterious, ROS serve important roles in cellular signaling pathways such as those involved in metabolism, differentiation, and immunity [82]. Indeed, non-cytotoxic concentrations of ROS foster mitochondrial homeostasis in a process known as mitohormesis [83]. In this scenario, certain stressors, such as physical exercise or hypoxia, increase mitochondrial ROS production which then activates the host’s antioxidant systems. Antioxidant defenses can then limit the concentrations of reactive metabolites, even in the face of increased mitochondrial activity [84,85,86]. Focusing on adipose tissue specifically, H_2_O_2_ promotes adipocyte lipid accumulation in vitro [87]. Physiological concentrations of ROS amplified cAMP-induced p38 mitogen-activated protein kinase activation in BAT, which promoted UCP1 expression after a cold challenge in mice [10]. In contrast, scavenging ROS with antioxidants or overexpression of Sestrin2, a stress-induced protein, reduce *Ucp1* expression and thermogenesis [10]. Moreover, it has been demonstrated in other tissues that ROS can oxidize cysteine residues in protein kinase A type I regulatory subunit I, which leads to the dissociation of the protein kinase A holoenzyme complex [88]. While still poorly defined, it may be possible that fat tissue is affected by these ROS-induced modifications given the importance of protein kinase A to lipolysis [89]. Although important to many homeostatic pathways in adipocytes, an excess of reactive metabolites can damage lipids, proteins, and DNA [82,90,91,92]. Thus, counterregulatory measures must be in place in order to maintain homeostasis given their potentially detrimental nature.

### 3.2. Antioxidant Defenses

Mammals rely on an array of enzymatic and non-enzymatic antioxidants to defend against, prevent, and reverse ROS and RNS effects. Enzymatic forms can be divided into primary or secondary molecules, depending on their ability to directly neutralize reactive metabolites [93]. The former contains some of the most effective antioxidant enzymes, including superoxide dismutase (SOD), glutathione peroxidase (GPx), and catalase. Secondary enzymatic antioxidants include glutathione reductase and glucose 6-phosphate dehydrogenase [93].

Non-enzymatic antioxidants can be derived endogenously or exogenously. Examples of endogenous non-enzymatic antioxidants include glutathione (GSH) and Q. Exogenous molecules include various vitamins and minerals, such as vitamin E and selenium [93]. Antioxidants most relevant to thermogenic adipose tissues are described in more detail below (Table 2).

#### 3.2.1. Enzymatic Antioxidants

Managing O_2_^●−^ is of the utmost importance to adipocyte energy regulation as it is the initial ROS formed by the ETC. Thus, SOD is an antioxidant of key interest in thermogenic fat [94]. Humans express three isoforms of SOD: SOD1 (cytosolic copper-zinc SOD; CuZn-SOD), SOD2 (mitochondrial manganese SOD; Mn-SOD), and SOD3 (extracellular CuZn-SOD) [105]. When considering all three collectively, the term “total SOD” is utilized. This enzyme carries out its action when O_2_^●−^ produced during electron leakage is dismutated to H_2_O_2_ and molecular oxygen [93]. The hydrogen peroxide produced can then be handled through various pathways. On the one hand, GPx is capable of reducing H_2_O_2_ to water or reducing organic peroxides (ROOH) to alcohols. On the other hand, catalase degrades H_2_O_2_ into water and molecular oxygen [93]. Given that H_2_O_2_ is a small, diffusible molecule that readily participates in signaling pathways such as inhibiting lipolysis, the actions of GPx and catalase can have profound effects on adipose redox reactions [75].

The aforementioned primary enzymatic antioxidants are important because they directly target ROS, thereby altering the immediate effects reactive metabolites can have on energy homeostasis. However, secondary enzymatic antioxidants are crucial in reestablishing molecules that are utilized in redox reactions. For instance, glutathione reductase is responsible for reducing oxidized glutathione (GSSG) to GSH while glucose 6-phosphate dehydrogenase replenishes NADPH; however, neither directly neutralize ROS, hence the classification as a secondary antioxidant enzyme [93].

Dramatic changes in enzymatic antioxidants can occur when NST is stimulated. For example, 3 h acute cold exposure decreased the mRNA expression of all SOD isoforms in the BAT of lean mice [95]. This effect was recapitulated in rats kept at 4 °C after 1 day of a 45-day cold exposure. Furthermore, total SOD activity was decreased over the entire study period [94]. Spasic and coauthors, however, found that CuZn-SOD activity was decreased during a 6 h cold exposure, while the Mn-SOD was elevated compared to rats kept at 22 °C. The authors also found that GPx activity was increased at 35, 75, and 105 days of 4 °C exposure [97]. The seemingly contrasting results regarding SOD may be related to the fact that mRNA expression does not necessarily correlate to protein activity. It is also apparent that the length of cold exposure has a strong influence over antioxidant dynamics in rodent BAT, which may also explain the divergent results. For instance, rats exposed to cold for 5 months had an increase in BAT SOD activity [97]. The catalase activity of rats from the same study was not elevated after 35 days, but was substantially higher at days 75 and 105 of the study compared to those kept at 22 °C [97]. Glutathione reductase was only increased after 105 days of being kept at 4 °C [97].

Selenoprotein P, a molecule capable of direct antioxidant actions and indirect effects by donating selenium, is downregulated during cold exposure in humans. This protein resulted in increased GPx4 and decreased heat production in primary brown adipocytes. Knocking out GPx4 in the cells restored their ability to respond to norepinephrine [11]. Furthermore, mice deficient in selenoprotein P in a knockout model had increased UCP1 activity after 2 h of cold exposure compared to the wild type controls. Thus, BAT may downregulate selenoprotein P as a means to increase norepinephrine sensitivity [11]. Collectively, this suggests that enzymatic antioxidants adapt to the shifts in oxidants, becoming more or less active during times of NST, which may help the adipocyte maintain homeostasis.

#### 3.2.2. Non-enzymatic Antioxidants

Glutathione turnover is one of the major endogenous antioxidant systems responsible for maintaining redox homeostasis in all cell types [106]. This molecule donates its hydrogen to ROS with the assistance of GPx, generating less-reactive molecules. For instance, GSH donates electrons to GPx4, which is responsible for neutralizing H_2_O_2_ produced by SOD and lipid peroxides that are formed during ROS-mediated lipid damage [107]. During these reactions, two GSH will dimerize to form GSSG. Another important non-enzymatic antioxidant to thermogenic adipose tissue is Q. As one of the mobile elements of the ETC, Q plays a central role in BAT utilizing fat to generate ATP and to produce heat [48,99].

Numerous vitamins and minerals are widely accepted to have antioxidant functions. Lipid-soluble vitamins A, D, and E, for example, accumulate in adipose tissue and can have antioxidant effects [100,108,109,110]. Vitamin A (particularly its metabolite, retinoic acid) primarily acts as an indirect antioxidant by regulating gene transcription related to redox balance [111]. Vitamin D also is an indirect antioxidant, altering the expression of other antioxidants such as glucose 6-phosphate dehydrogenase and GPx [112]. Vitamin E and selenium are amongst the most commonly referenced, and both are derived exogenously from dietary sources. The form of vitamin E considered the most biologically active is α-tocopherol; thus, it is highly encountered throughout the literature [113,114]. This antioxidant donates its hydrogen to lipid peroxides, thereby disrupting the ROS-mediated oxidation reactions in cellular membranes [115]. Selenium, on the other hand, is an important cofactor for other antioxidants utilized in adipose redox mechanisms, including certain GPx [103].

As with the enzymatic antioxidants, non-enzymatic antioxidants show dynamic changes during conditions that enhance NST. During acute cold exposure, the BAT glutathione metabolism was one of the most altered pathways [116]. In a different study, GSSG was increased significantly in BAT when mice were subjected to increasing percentages of calorie restriction [117]. Rats exposed to 4 °C for 105 days had significantly higher GSH in BAT than those kept at 22 °C for the same amount of time [97]. Vitamin E was not significantly different between rats kept at 22 °C or 4 °C after 35, 75, or 105 days [97]. However, Tanaka-Yachi and colleagues noted that *Ucp1* mRNA expression was increased approximately 2-fold in 3T3-L1 adipocytes treated with α-tocopherol compared to untreated cells. Furthermore, mitochondrial content and UCP1 protein expression were also enriched in the α-tocopherol group [102]. The results presented by Tanaka-Yachi and coauthors suggest that vitamin E analogs may be important for differentiating thermogenic adipocytes. The vitamin A metabolite, retinoic acid, promoted a brown adipocyte phenotype in WAT by encouraging angiogenesis [100]. Vitamin D, on the other hand, tended to decrease uncoupling protein expression in activated rat brown adipocytes [101].

### 3.3. Additional NST-Specific Redox Considerations

Currently, there is an intense debate over whether uncoupling proteins, themselves, have roles in ROS mitigation, which has been recently reviewed elegantly elsewhere [118,119]. Moreover, as redox reactions critical to BAT function rely on a balance between reactive metabolites and antioxidants, it does not do well to consider only the former or the latter in isolation. Indeed, they both should be evaluated together and considered in light of other factors, such as which depot is being investigated and whether any other physiological stressors are present. For instance, different adipose depots demonstrate varying degrees of oxidant and antioxidant potentials, which are further altered by a lean versus obese phenotype [9]. The brown adipose tissue of lean rats had the highest Complex IV activity, which can be used as a proxy for oxidative potential, compared to inguinal and epididymal white adipose [9,120]. Furthermore, Mn-SOD, coenzyme Q9 (CoQ9), Q, and α-tocopherol were the highest in lean BAT [9]. Collectively, this suggests that lean BAT has the highest oxidative potential but also seemingly has the appropriate defenses to counterbalance the oxidation necessary for its metabolic function. Compared to lean BAT, obese rats had significantly less Complex IV, indicating a reduced mitochondrial metabolism. Additionally, these rats had less glutathione, CoQ9, and Q, but higher catalase activity [9]. Considered collectively, changes in antioxidant responses during various physiological processes such as lipolysis and NST clearly show that mammals demonstrate a remarkable ability to adapt to variable oxidative environments. In fact, disturbances to the oxidant–antioxidant balance can result in numerous pathologies, some of which are described below.

## 4. Manipulating Adipose Redox Mechanisms as Potential Metabolic Disease Therapies

Dysregulated energy metabolism pathways driven by redox reactions are major underlying components of metabolic diseases such as diabetes and FLD [4,5,121]. Indeed, imbalances between ROS and antioxidants have been widely reported for both diseases [122,123,124]. For instance, the adipocyte deletion of GPx4 in mice resulted in metabolic disturbances, such as decreased glucose tolerance and insulin sensitivity, even in the absence of other metabolic challenges such as a high-fat diet [125]. Given the importance of redox reactions to adipocyte energy regulation and NST, altering oxidants, antioxidants, or both presents an opportunity to modulate energy metabolism and metabolic diseases.

### 4.1. Current Therapeutics Directed towards Thermogenesis

The content of BAT in humans has been associated with improved cardiometabolic health, and the benefits seemed greater in individuals who were obese [14]. Hence, a particularly exciting target for metabolic disease modulation has been thermogenic fat due to its unique ability to dissipate energy. As such, many studies have investigated various mechanisms that act as NST agonists. Triggering adult thermogenic fat can be accomplished through methods that stimulate β3-adrenergic pathways, including cold exposure, dietary alterations (e.g., high-calorie diets, supplements), and pharmacological intervention [49,126]. Amongst the molecules that can activate NST, β3-adrenergic agonists such as CL316243 are commonly referenced throughout the literature. Earlier studies using a model of diet-induced obesity established that when treated subcutaneously with CL316243, rat BAT had markedly increased UCP1 content. Furthermore, the WAT of rats with diet-induced obesity exhibited an acquisition of a brown-like phenotype, including metabolic rates 40–45% higher than controls and increased UCP protein [127]. Regardless of how NST was activated, similar findings have been demonstrated in numerous other studies as well, indicating that the stimulation of thermogenic fat may be a suitable treatment for metabolic diseases [128]. Although it is not discussed further herein, skeletal muscle is abundant throughout the body and also performs NST. Therefore, muscle NST may be a target for metabolic disease modulation [62].

Although the activation of thermogenic fat has shown promise in rodent models, analogous effects are not always noted in humans [126,129]. This may be due in part to a paucity of BAT activity in obese individuals compared to their lean counterparts, or the differences in the thermoregulation between mice and humans [130,131,132,133]. Alternatively, it has been demonstrated that β1-adrenergic receptors are involved in human BAT UCP1 expression, not β3 [134]. Moreover, NST-activating treatments may have adverse side effects. For instance, using the pharmacological uncoupler 2, 4-dinitrophenol to mimic UCP1 effects can lead to altered respiration in all cells at high doses. Ultimately, this can lead to hyperthermia and the death of patients [135]. If non-specific β-adrenergic agonists are employed, off-target effects on the cardiovascular system, such as increased heart rate, may occur [136]. Even mirabegron, a β3-specific drug, can result in heart failure due to its effects on the cardiovascular system [16]. As a result, there currently is not a thermogenic regulator available on the market to use in the clinic [137]. However, as discussed below, targeting intrinsic redox reactions present in thermogenic adipose tissue may represent an underexplored therapeutic option for increasing NST and treating metabolic diseases.

### 4.2. Current Use of Antioxidants to Combat Metabolic Disease

Traditionally, antioxidant supplementation is proposed as a part of the therapeutic strategy for pathologies associated with redox dysregulation. Diabetes and FLD are no exception, with numerous studies and reviews discussing the matter [138,139,140,141]. Decreased serum concentrations of selenium and GPx have been reported in diabetic patients compared to healthy controls [142]. Similarly, the total antioxidant capacity was reduced in the plasma of FLD patients [143]. Thus, antioxidant supplementation would be a logical solution to remedy metabolic disease. Despite the popularity of this treatment option, evidence of clinical success is sparse when considering any molecule as a standalone therapy [138]. For instance, Wang and coauthors found that there was a 3–6% decrease in body fat percentage with every 1 μg/kg/d increase in dietary selenium intake in a population-based study [144]. However, in another study completed by Hawkes and Keim, high dietary selenium supplementation (297 μg/d) resulted in weight gain in healthy men, while low supplementation (14 μg/d) lead to weight loss [145]. Given the wide range of variability between studies, such as supplementation dose, measured outcomes, and experimental procedures to collect data, it is not surprising that reaching a consensus on the benefit of antioxidants has been challenging. Therefore, a greater understanding of redox pathways and their role in contributing to metabolic disease is needed.

### 4.3. New Perspectives to Altering the Redox Balance in Thermogenic Adipose Tissue as Metabolic Disease Therapies

The works presented thus far support the concept that an oxidative environment is supportive for NST. Thus, excess antioxidant supplementation may be counterproductive in activating thermogenic fat. Indeed, it may be more desirable to increase particular intrinsic redox pathways to activate BAT [6,81]. The deletion of adipocyte Mn-SOD increased energy expenditure and fatty acid oxidation along with decreased body weights when fed a high-fat diet [73]. Mitochondrial biogenesis and uncoupling were increased specifically in the inguinal WAT of these mice, suggesting the development of a beige phenotype. Finally, knocking out Mn-SOD in the adipocytes resulted in 40% less circulating free fatty acids, prevented lipid accumulation in the liver, and prohibited diet-induced glucose and insulin intolerance during high-fat feeding in the mice [73]. The depletion of GSH resulted in enhanced BAT thermogenesis and the conversion of white adipocytes to a beige phenotype [98]. Concentrations of GSH can be decreased with substances that inhibit their synthesis such as buthionine sulfoximine (BSO) [146]. Mice treated with BSO for 24 h had reduced fat mass compared to untreated mice, suggesting the activation of energy expenditure pathways [98]. Indeed, the expression of genes associated with thermogenesis, including *Ucp1*, were upregulated with the BSO treatment in BAT. The upregulation of NST-associated genes was even more prominent in epididymal WAT [98]. Unlike other agents that show promise in rodents but fail to demonstrate safe, effective changes in humans, BSO has been tested in phase I clinical trials for cancer without causing tissue toxicity [147].

In some cases, however, antioxidant supplementation may encourage a white-to-beige phenotype in adipocytes. Male mice fed a high-fat diet concurrently with supplements composed of mitochondrial enhancers and antioxidants (beetroot extract, Q, alpha lipoic acid, vitamin E, creatine, green tea extract, black tea extract, green coffee bean extract, conjugated linoleic acid, and forskolin) had increased mitochondrial content and evidence of a phenotypic switch in WAT compared to mice fed only the high-fat diet [148]. Interestingly, the UCP1 protein content was not altered in the BAT of these mice, despite the supplemented groups having an increased expression of genes related to mitochondrial function, energy homeostasis, and ROS defense compared to high-fat diet-only mice [148]. While the contribution of each ingredient to the browning effect was not determined in this study, it did provide evidence that increasing the dietary intake of these substances can have beneficial metabolic effects and may be a worthy adjunct to current metabolic disease therapies.

Antioxidants may directly promote BAT function as well. Recently, Jedrychowski et al. demonstrated that 2.25 ppm selenium supplementation in the diet for 8 wk enhanced UCP1 cysteine 253 selenation, which subsequently elevated NST in the BAT and beige adipose following CL316243 stimulation. These mice were also resistant to weight gain while being fed a high-fat diet [104]. Moreover, this UCP1 selenation site was important for preventing adipose tissue redox stress and inflammation in mice [149]. Thus, supplementing selenium could benefit metabolic health if given within an appropriate dose range. Of importance, however, is that the ability to alter the redox balance exclusively in adipose tissue is unknown. Therefore, each of the aforementioned potential interventions would have a whole-body effect and may impact redox reactions elsewhere. Overall, redox mechanisms may serve as an underexplored avenue of modulating NST. Further elucidating the complexities in these pathways and how they are altered during NST can reveal novel targets for metabolic disease therapies.

## 5. Conclusions and Future Perspectives

Adipose tissue plays a fundamental role in maintaining whole-body energy homeostasis due to its ability to balance energy storage and release in a sophisticated manner. The potential to mitigate metabolic diseases by activating thermogenic fat, thus enhancing energy expenditure, has become an area of intense research focus recently. However, while promoting NST to assuage diseases such as diabetes and FLD holds promise in rodents, many effects do not translate to humans. Therefore, it is important to elucidate the analogous mechanisms underlying thermogenic pathways between research models and human patients so that proper therapeutics can be developed.

Several redox mechanisms regulate NST in thermogenic fat, thereby representing a pool of potential pathways to be targeted to mitigate metabolic diseases. Manipulating redox reactions through the alteration of antioxidant availability is not a novel concept. Often, however, it is assumed that bolstering antioxidant systems or antioxidant supplementation will be beneficial to an organism. Thermogenesis exemplifies how reactive metabolites produced from redox reactions are not inherently problematic and can actually serve a beneficial role. Indeed, in the case of energy dissipation by brown and beige adipocytes, the metabolically favorable option may be to avoid excessive ROS scavenging by antioxidants. However, it should not be forgotten or understated that redox balance is extremely complex. Thus, purposefully inhibiting antioxidants to support one pathway (e.g., NST) may be detrimental to another. This is especially pertinent considering that there is not currently a way to manipulate redox mechanisms exclusively in thermogenic adipose, meaning whole-body changes may occur with any dietary or pharmacological intervention to alter the redox balance. Ultimately, it will be necessary to unravel more details about the delicate balance of adipose redox mechanisms so that more targeted therapies can be developed.

## Figures and Tables

**Table 1:** Reactive metabolites associated with thermogenic adipose tissue.

Reactive Metabolite	Association with Thermogenic Fat	Source
Superoxide (O_2_^●−^)	Increases in vivo after cold exposure	[71]
	Enhances UCP1 ^1^ proton transport	[72]
	Stimulates mitochondrial uncoupling and fatty acid oxidation	[73]
	Promotes beige phenotype in WAT ^1^	[73]
Hydrogen peroxide (H_2_O_2_)	Increases in vivo after cold exposure	[71]
	Decreases mitochondrial membrane potential	[74]
	Inhibits lipolysis	[75]
Nitric oxide (NO^●^)	Increases blood flow following catecholamine stimulation	[76]
	Increases BAT ^1^ mass and UCP1 content	[77]

^1^ UCP1 = uncoupling protein 1; WAT = white adipose tissue; BAT = brown adipose tissue.

**Table 2:** Antioxidants relevant during non-shivering thermogenesis (NST).

Antioxidant	Association with Thermogenic Fat	Source
Superoxide dismutase (SOD)	mRNA expression and antioxidant activity decrease after acute and chronic cold exposure	[94,95]
Glutathione peroxidase (GPx)	Upregulation of GPx4 by selenoprotein P promotes resistance to norepinephrine-induced NST ^1^	[11]
Catalase	Deficiency leads to enhanced fatty acid shuttling to BAT ^1^, induces NST	[96]
	Activity increases after prolonged cold exposure in BAT	[97]
Glutathione reductase	Increases in BAT after chronic cold exposure	[97]
Selenoprotein P	mRNA downregulates in BAT after acute cold exposure; negatively correlates with human BAT activity	[11]
Sestrin2	Decreases *Ucp1* ^1^ mRNA; decreases NST after cold exposure	[10]
Glutathione (GSH)	Increases in BAT after chronic cold exposure	[97]
	Decreases during white-to-brown phenotype conversion after acute cold exposure and β3-adrenergic agonist stimulation; mRNA levels of *Ucp1* upregulate in BAT and WAT ^1^	[98]
Ubiquinone (Q)	Electron carrier in the mitochondrial electron transport chain	[67]
	Uptake into BAT required for normal NST	[99]
Vitamin A	Retinoic acid promotes a brown adipocyte phenotype in WAT	[100]
Vitamin D	Decreases uncoupling protein expression in rat brown adipocytes	[101]
Vitamin E	Promotes a beige phenotype	[102]
Selenium	Cofactor for many antioxidants relevant to NST, including GPx	[103]
	Improves UCP1 efficiency in BAT	[104]

^1^ NST = non-shivering thermogenesis; BAT = brown adipose tissue; UCP1 = uncoupling protein 1; WAT = white adipose tissue.
